# Supplementary material for: NNC 26-9100 increases Aβ1-42 phagocytosis, inhibits nitric oxide production and decreases calcium in BV2 microglia cells
Source: PLoS One. 2021 Jul 8;16(7):e0254242. doi: 10.1371/journal.pone.0254242 (PMC8266108; doi:10.1371/journal.pone.0254242)
Supplement: Attachment — Submitted filename: Responses to Reviewers.docx [file pone.0254242.s001.docx]

We thank the Reviewers for their comments. Each of the text changes in the manuscript are highlighted by underlines.

**Responses to Reviewers**

**Reviewer #1:** The manuscript examines the effect on SST4 receptor agonist NNC 26-9100 on BV2 microglia cells with special focus on cell function related to Alzheimer’s disease. The design of the study is elaborate. Methods are modern and English language is excellent. I have two minor questions:

Why is the maximal concentration of NNC 26-9100 different in Alamar blue, LDH and nitrite assays compared to the other experiments?

Author Response- In the 96-well plate assays (alamar blue, nitrite, and lactate dehydrogenase), we explored a range of concentrations (0, 0.104, 0.208, 0.417, 0.833 μM). The NNC compound had a maximal effect in the nitrite assay at 0.833 μM, which decreased LPS-induced nitrite to near baseline. The flow cytometry-based assays (sytox, cell scattering, cell calcium and Aβ1-42 uptake) are sensitive but low throughput, so we choose just one concentration to forward. We choose 1.0 μM, a concentration marginally above 0.833 μM, and likely to show maximal effect without cell toxicity. We included this text in the Discussion section of the revised manuscript.

They authors conclude on shift of microglia to M2 phenotype. This should only be included if respective cell markers are examined and phenotype shift can be objectively demonstrated.

Author Response- We agree with the reviewer. The sentence referring to M2 phenotype in the last paragraph in the Discussion was removed.

**Reviewer #2**: The well-written manuscript of Schober and co-authors presents a nice and thorough work, analyzing the effects of an SSTR4 agonist on microglia function. The Authors confirmed that NNC is able to decrease the LPS-induced lactate dehydrogenase and nitrite release, while in the absence of LPS, increase Aβ uptake. The data presented in this manuscript confirm the previous results of the Authors. In my opinion, the results are clear and well-described, only few modifications are needed:

The explanations of some abbreviations are missing from the manuscript (SSTR4, SRIF).

Author Response- The abbreviations are now defined at their first usage.

The article would greatly benefit from a brief description of the role of SSRTs and SRIF in AD, to emphasize the potential therapeutic applicability of the results.

Author Response- A section in the Discussion beginning was added which points the reader to references on the topic of somatostatin and somatostatin receptors in AD.
